# Fast Healthcare Interoperability Resources (FHIR)–Based Quality Information Exchange for Clinical Next-Generation Sequencing Genomic Testing: Implementation Study

**DOI:** 10.2196/26261

**Published:** 2021-04-28

**Authors:** Donghyeong Seong, Sungwon Jung, Sungchul Bae, Jongsuk Chung, Dae-Soon Son, Byoung-Kee Yi

**Affiliations:** 1 Department of Health Sciences and Technology Samsung Advanced Institute for Health Sciences & Technology Sungkyunkwan University Seoul Republic of Korea; 2 Smart Healthcare Research Institute Samsung Medical Center Seoul Republic of Korea; 3 Samsung Genome Institute Samsung Medical Center Seoul Republic of Korea; 4 School of Big Data Science Data Science Convergence Research Center Hallym University Chuncheon Republic of Korea; 5 Department of Digital Health Samsung Advanced Institute for Health Sciences & Technology Sungkyunkwan University Seoul Republic of Korea

**Keywords:** FHIR, clinical NGS genomic testing, clinical massive parallel sequencing, quality control, genomic reporting

## Abstract

**Background:**

Next-generation sequencing (NGS) technology has been rapidly adopted in clinical practice, with the scope extended to early diagnosis, disease classification, and treatment planning. As the number of requests for NGS genomic testing increases, substantial efforts have been made to deliver the testing results clearly and unambiguously. For the legitimacy of clinical NGS genomic testing, quality information from the process of producing genomic data should be included within the results. However, most reports provide insufficient quality information to confirm the reliability of genomic testing owing to the complexity of the NGS process.

**Objective:**

The goal of this study was to develop a Fast Healthcare Interoperability Resources (FHIR)–based web app, NGS Quality Reporting (NGS-QR), to report and manage the quality of the information obtained from clinical NGS genomic tests.

**Methods:**

We defined data elements for the exchange of quality information from clinical NGS genomic tests, and profiled a FHIR genomic resource to enable information exchange in a standardized format. We then developed the FHIR-based web app and FHIR server to exchange quality information, along with statistical analysis tools implemented with the R Shiny server.

**Results:**

Approximately 1000 experimental data entries collected from the targeted sequencing pipeline CancerSCAN designed by Samsung Medical Center were used to validate implementation of the NGS-QR app using real-world data. The user can share the quality information of NGS genomic testing and verify the quality status of individual samples in the overall distribution.

**Conclusions:**

This study successfully demonstrated how quality information of clinical NGS genomic testing can be exchanged in a standardized format. As the demand for NGS genomic testing in clinical settings increases and genomic data accumulate, quality information can be used as reference material to improve the quality of testing. This app could also motivate laboratories to perform diagnostic tests to provide high-quality genomic data.

## Introduction

Next-generation sequencing (NGS) technology has been rapidly adopted in clinical practice, with the scope extended to early diagnosis, disease classification, and treatment planning [1-3]. To implement clinical NGS applications, health care providers operate their own NGS laboratories or request genomic tests to external laboratories. The NGS genomic testing reports provide information regarding genomic variations and related data. However, different templates and data elements are used depending on the laboratory, and most reports are provided in text or PDF format [4,5]. As the number of requests for NGS genomic testing increases, considerable efforts have been made to deliver the testing results clearly and unambiguously [6-10]. Moreover, international standards development organizations have developed reporting standards such as the Fast Healthcare Interoperability Resources (FHIR) Genomics Reporting Implementation Guide and International Organization for Standardization (ISO) standards to exchange complex clinical genomic data and interpretations [11-14].

For the legitimacy of clinical NGS genomic testing, the quality information from the process of producing the genomic data should be included within the test results [15-17]. The demand for genomic testing of small tissue samples and needle biopsies is increasing [18], and it remains challenging to determine the reliability of the genomic test results. When testing small quantities or low-quality samples, including low-purity specimens and formalin-fixed paraffin-embedded (FFPE) specimens, quality information can be considered for the interpretation of diagnostic results as evidence. Confirming that there is no specific variation is a particular challenge, especially without quality information. For the validity and utility of clinical NGS genomic testing, the US Centers for Medicare and Medicaid Services regulates all laboratory tests performed on humans through the Clinical Laboratory Improvement Amendments, and the US Food and Drug Administration (FDA) requires information regarding the clinical validity for genomic tests [19,20]. In Korea, the Ministry of Food and Drug Safety (MFDS) has initiated the clinical laboratory accreditation program since 2017 [21]; however, this provides accreditation of the entire NGS process rather than the reliability of individual samples. When a clinician performs NGS genomic testing for diagnostic purposes, quality information can be used to interpret the results for individual samples. In addition, when constructing a reference database using these samples, it is essential to include this quality information.

The successful practice of precision medicine depends on clinical genomic data sharing and knowledge-based interpretations of genomic variant data at the point of care [11]. To improve interoperability as part of precision medicine, health care stakeholders encourage the use of application programming interfaces (APIs) and app-based ecosystems such as SMART on FHIR, CDS Hooks, and SMART Markers [22-26]. These platforms enable easy implementation for health care use cases and facilitate functional extensibility. There are several apps based on clinical genomics use cases on the SMART on FHIR platforms, such as the SMART Precision Cancer Medicine and SMART Cancer Navigator apps [27-29].

In this study, we developed the NGS Quality Reporting (NGS-QR) app to exchange the quality information of clinical NGS genomic testing. To exchange the information in a standardized format, we profiled a FHIR genomic resource based on ISO/TS 22692:2020 Genomics Informatics-Quality Control Metrics for DNA sequencing, which defines the quality-related data for the entire NGS process, including sample preparation, library preparation, sequencing, and data processing [15]. This app enables the performance comparison of clinical NGS genomic testing and monitoring of the quality status of the current sample as the data accumulate.

## Methods

### Overview

This study describes the development of the NGS-QR app, a FHIR-based web app, to report, manage, and monitor the quality information from the process of producing genomic data. As shown in Table 1, the development was composed of the following phases: (1) requirement analysis, (2) design, (3) implementation, and (4) testing. In the requirement analysis phase, we defined use cases and selected data elements such as DNA purity and integrity, library input amount and size, and sequencing running quality, in accordance with standards and guidelines. In the design phase, we profiled a FHIR resource on the existing genomic resource to exchange quality information in a standardized manner. We also designed user interfaces and functions for the NGS-QR app. In the development phase, we developed the three following components: web app (NGS-QR app), FHIR server, and R Shiny server. The web app includes a FHIR resource handler to generate and parse FHIR resources. Finally, in the testing phase, the NGS-QR app was validated using real-world data collected from the NGS pipeline, a targeted sequencing platform.

**Table 1:** Overview of development of the next-generation sequencing-quality reporting (NGS-QR) app.

Development phase		Description
**Requirement analysis**		
	Use cases	Define use cases for quality information exchange of clinical NGS^a^ genomic testing
	Data elements	Select data elements in accordance with standards and guidelines (eg, ISO^b^, US FDA^c^, ACMG^d^)
**Design**		
	FHIR^e^ genomic profile	Profile a FHIR resource on the existing genomic resource, MolecularSequence
	System components	Design user interfaces and functions of the NGS-QR app
**Implementation**		
	Web app (NGS-QR app)	Develop user interfaces and functions of the web app; develop a FHIR resource handler to generate and parse FHIR resources
	FHIR server	Develop a FHIR server and repository; apply the FHIR QcMetrics profile to the FHIR server
	R Shiny server	Develop R code for statistical analysis of NGS experimental data
**Testing**		
	Data collection	Collect real-world data generated from the NGS pipeline
	Data exchange	Exchange real-world data between the FHIR server and NGS-QR app using FHIR APIs^f^
	Quality management	Check the quality information of NGS genomic testing using the dashboard and statistical analysis

^a^NGS: next-generation sequencing.

^b^ISO: International Organization for Standardization.

^c^US FDA: US Food and Drug Administration.

^d^ACMG: American College of Medical Genetics.

^e^FHIR: Fast Healthcare Interoperability Resources.

^f^APIs: application programming interfaces.

### Use Cases

**Figure 1:**
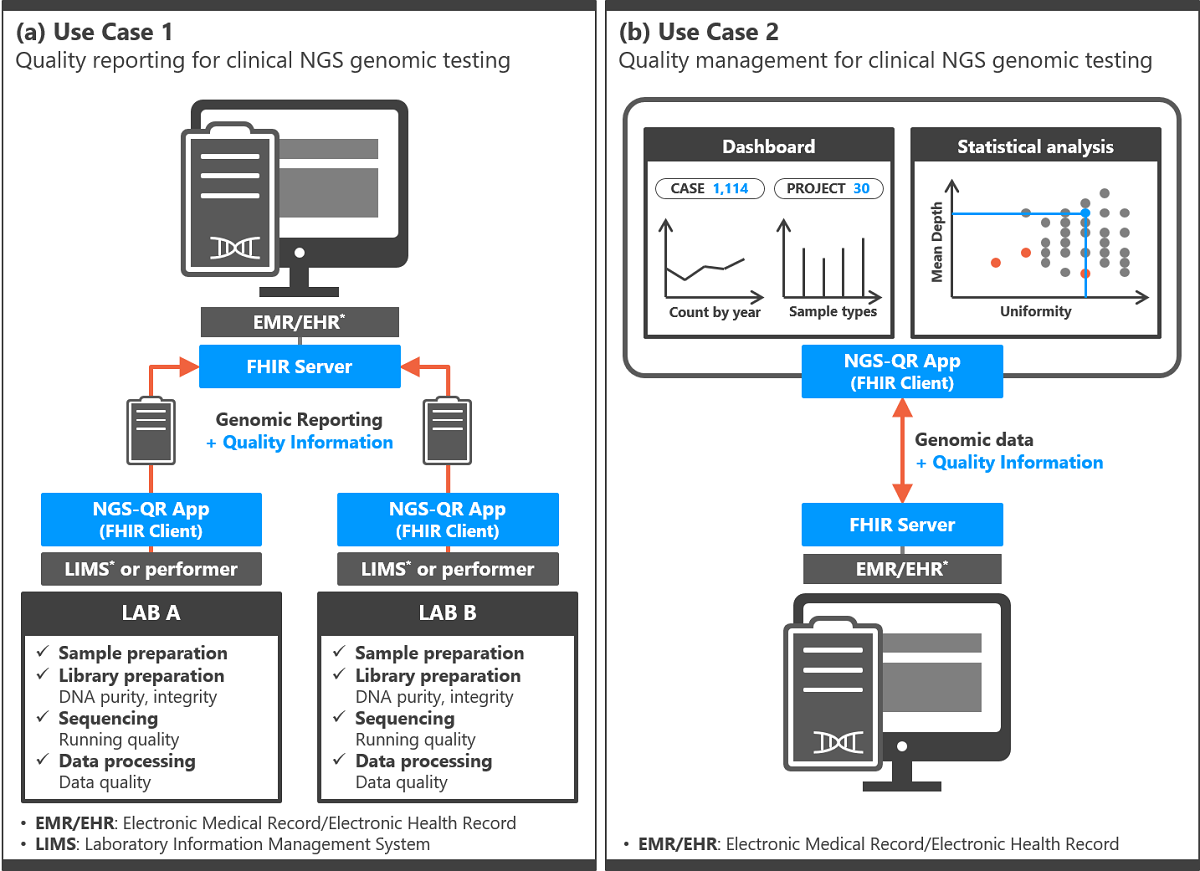
Use cases for exchanging the quality information of clinical next-generation sequencing (NGS) genomic testing. (a) Quality reporting for clinical NGS genomic testing. (b) Quality management for clinical NGS genomic testing. FHIR: Fast Healthcare Interoperability Resources.

We defined two use cases: quality information reporting and quality management for clinical NGS genomic testing. The first use case is related to the quality information reporting for clinical NGS genomic testing, as shown in Figure 1a. The laboratory reports the results of NGS genomic testing to the hospital along with quality information. This use case assumes that the hospital information system provides the FHIR APIs. The laboratory requested for the NGS genomic testing sends the results using the NGS-QR app, which acts as an FHIR client. When the user fills out the reporting form in the NGS-QR app, a FHIR resource is created in JSON format and then the resource is sent to the server via the FHIR API. Hospitals that request NGS genomic testing to multiple laboratories can receive information in the same format through this standardized method.

The second use case is related to the quality management for clinical NGS genomic testing, as shown in Figure 1b. Users such as health care providers and performers of NGS testing manage and monitor the quality status of the genomic data received from the laboratories. Through the NGS-QR app, users can retrieve experimental data from the FHIR server and view the summary such as the total number of genomic tests and the number of tests based on the year or specimen type for all genomic test results. Moreover, quality information such as DNA purity, integrity, and data quality for each sample can be compared through statistical analysis. This step can be used to determine the reliability of clinical NGS genomic testing.

### Data Elements Selection

The NGS workflow involves complex procedures consisting of several steps, which are broadly divided into sample preparation, library preparation, sequencing, and data processing. In this process, various types of experimental conditions and results are generated and captured. As shown in Table 2, we selected data elements based on the ISO 22692 [15] and guidelines such as those of the FDA [20], American College of Medical Genetics [30], and College of American Pathologists [31] for quality management and comparative analysis. For this study, approximately 30 data elements were selected.

**Table 2:** Next-generation sequencing (NGS) workflow and data elements.

NGS workflow		Data elements
**Sample preparation**		
	Sample sequencing type	sequencing type, target gene
	Sample information	specimen type, sampling date
**Library preparation**		
	DNA extraction	DNA extraction kit, DNA purity (eg, OD^a^ 260/280, OD 260/230), DNA integrity (eg, DNA median size)
	Library construction	library input amount, library input size, library construction kit
**Sequencing**		
	Sequencing information	sequencing instrument, read length, sequencing direction, running mode
	Running quality	error rate, percent data quality (>Q30)
**Data processing**		
	Data quality	total reads, mean coverage, uniformity, on-target rate, Q30, PR^b^ score
	Sequencing alignment	mapping algorithm, sequencing alignment software
	Variant calling	variant calling software, quality score, allelic read percentage
	Variant filtering and annotation	germline filter criteria, reference database

^a^OD: optical density.

^b^PR: pass rate.

### FHIR Genomics Resource Profile

We profiled a FHIR genomic resource by defining constraints and extensions for exchanging quality information with a standardized method. Since the results of NGS genomic testing include genomic variant information and its quality-related data, the FHIR R4 MolecularSequence resource [32] was used for tailoring the NGS data to the use cases in this study. We created a QcMetrics element (a FHIR extension) in the resource and added data elements for each process under QcMetrics. Textbox 1 shows an example of a FHIR genomics resource that includes the QcMetrics profile. We uploaded the profile to the public FHIR registry SIMPLIFHIR.NET [33].

**Example of the Fast Healthcare Interoperability Resources genomic resource that includes the QcMetrics profile.**

{

“resourceType”: “MolecularSequence”,

“extension”: [

{

“url”: “http://example.org/fhir/StructureDefinition/QcMetrics”,

“extension”: [

{

“url”: “dnaExtraction”,

“extension”: [

{

“url”: “dnaExtractionKit”,

“valueString”: “qiagen allprep DNA/RNA mini kit”

},

{

“url”: “dnaPurity”,

“extension”: [

{

“url”: “od260280”,

“valueDecimal”: 2.1

},

{

“url”: “od260230”,

“valueDecimal”: 2.3

}]

},

{

“url”: “dnaIntegrity”,

“units”: “bp”,

“valueDecimal”: 60000

}]

},

…

}

### Implementation

**Figure 2:**
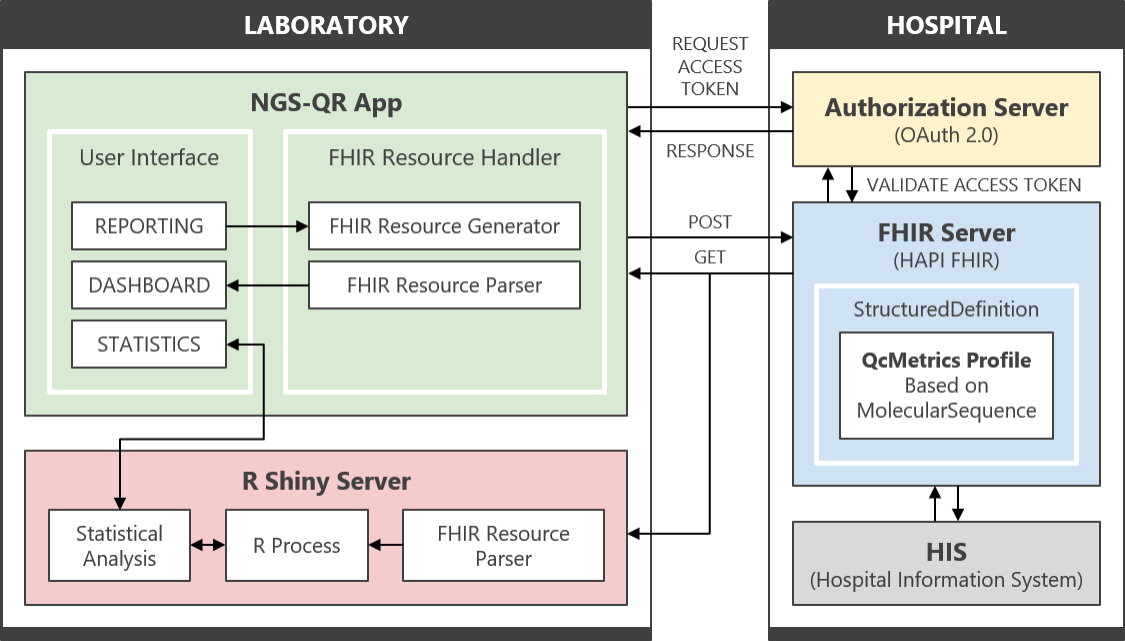
Concept model of the NGS-QR app, FHIR server, and R Shiny server. FHIR: Fast Healthcare Interoperability Resources.

We developed the following three major components: the NGS-QR app (FHIR client), FHIR server, and R Shiny server, as shown in Figure 2. The NGS-QR app was developed using Node.js (v12.13.1), which is composed of user interfaces (UIs), a FHIR resource handler, and a REST API module. The app has been deployed to the App Gallery of SMART on FHIR, which is an open platform for substitutable third-party health apps to connect to electronic medical record (EMR)/electronic health record (EHR) systems with appropriate security guarantees [34,35]. The source code of the app is available at GitHub [36]. The FHIR server was locally installed using the HAPI FHIR server (v4.0.0), which is an open-source Java implementation of the FHIR specification. The QcMetrics profile was added to the FHIR server. The source code for operating the R Shiny server was written in RStudio Cloud (R version 3.6.3) and the server was deployed to the R cloud platform, Shinyapp.io [37]. The R Shiny server fetched the data used for statistical analysis from the FHIR server. The R source code is available at GitHub [38].

FHIR has a set of security recommendations that identifies communications security, authentication, authorization, access control, and auditing [39]. We applied Transport Layer Security (TLS) and OAuth 2.0, which are industry-standard protocols for communications security and authentication [40,41]. The TLS was used for the encryption of FHIR resources transmitted between the NGS-QR app and the FHIR server. The OAuth 2.0 protocol was used to grant the NGS-QR app access to the FHIR server. The NGS-QR app requests an access token by authenticating with the Authorization Server. The Authorization Server authenticates the NGS-QR app and issues an access token. The access token was used for security credentials for the NGS-QR app to make API requests on behalf of a user. 

## Results

### Data Collection

Samsung Medical Center (SMC) has developed and utilized a cancer panel sequencing pipeline, namely CancerSCAN, to determine effective treatment methods for patients based on data from more than 15,000 panel sequencing studies since 2014 [17,42,43]. The SMC received clinical laboratory accreditation by the MFDS in 2017, and CancerSCAN is clinically used for the diagnosis and prognosis of cancer patients [17]. For this study, approximately 1000 data entries were collected from CancerSCAN. They contained the experimental conditions and results for FFPE specimens, fresh cells, and cell lines.

### User Interfaces and Functions

**Figure 3:**
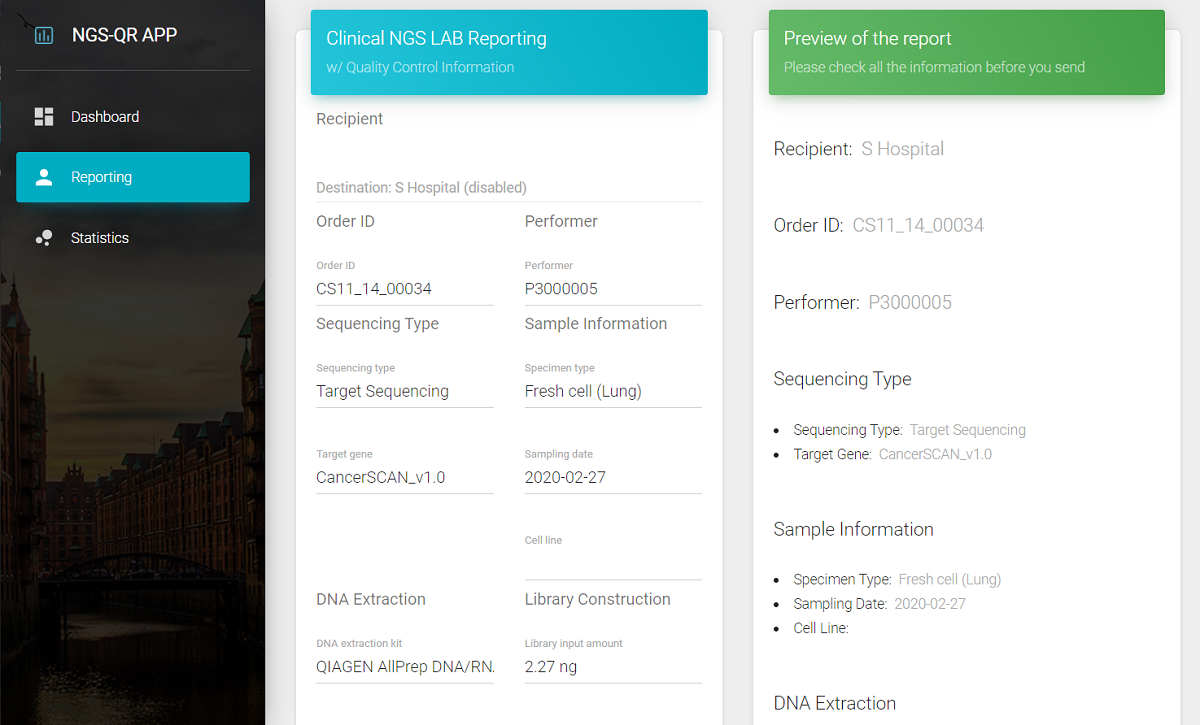
Screenshots of the user interface: reporting quality information of clinical next-generation sequencing (NGS) genomic testing.

**Figure 4:**
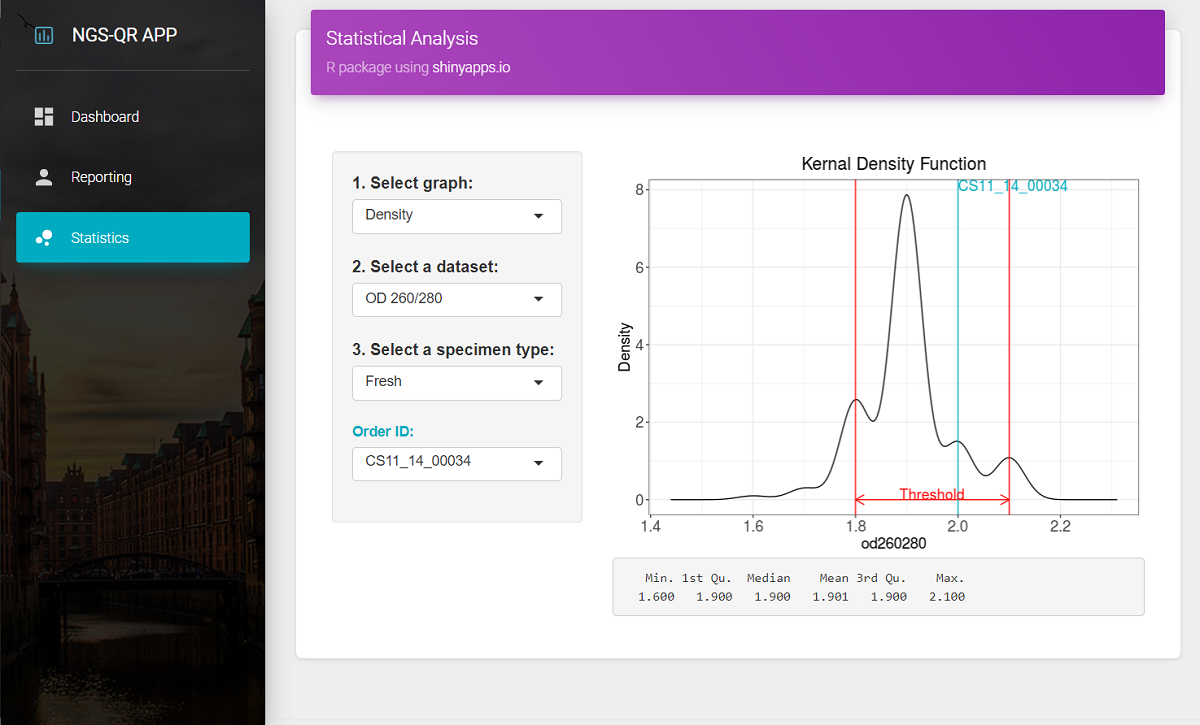
Screenshots of the user interface: quality management of clinical next-generation sequencing genomic testing.

In the reporting UI, input data are converted to a FHIR genomic resource in JSON format using the FHIR resource generator, and the resource is sent to the FHIR server using the POST method, as shown in Figure 3. In the dashboard UI, genomic resources in the FHIR server are compiled using the GET method to display the summary of data such as the total number of genomic tests and the number of tests based on the year and specimen type for the complete genomic test results. In the statistics UI, the user can select the group of samples, and view the distribution and threshold of each experimental parameter. The user can also check the current quality status of each sample in the overall distribution, as shown in Figure 4.

### Data Statistics

**Figure 5:**
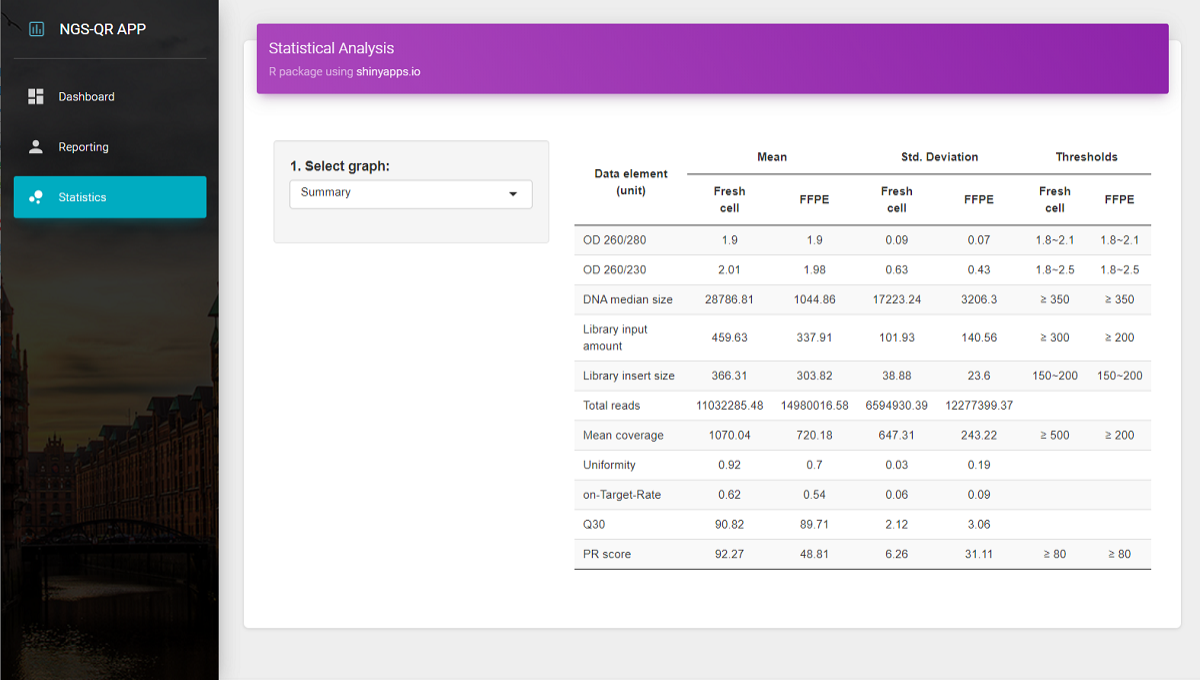
Statistics of the experimental data used in this study. FFPE: formalin-fixed paraffin-embedded; OD: optical density; PR: pass rate.

We validated the utility of the NGS-QR app using real-world data from the NGS pipeline CancerSCAN in SMC. All data were converted to FHIR genomic resources and sent to the FHIR server using the NGS-QR app. Figure 5 shows the statistics of the experimental data used in this study. As expected, fresh cell samples had better quality than FFPE samples in almost every category. 

## Discussion

### Principal Results

NGS technology has been widely adopted in clinical practice, which will play a prominent role in precision medicine. Although many countries and international institutions have developed NGS guidelines to implement clinical NGS applications, the quality criteria for determining the reliability of NGS genomic testing have not yet been standardized due to the complexity of the NGS technology. Since the scope and purpose of NGS applications are very diverse, it remains challenging to evaluate the validity and utility of clinical NGS genomic testing. At present, most individual laboratories have their own processes and criteria; thus, it is important to share quality information from the process of generating genomic data.

In this study, we propose the NGS-QR app to exchange quality information for clinical NGS genomic testing. This study provides the following main contributions to the field. First, we demonstrated that the quality information managed only by individual laboratories could be shared using a standardized format. In the NGS workflow, the experimental conditions change depending on the purpose of the tests and the status of the samples. Thus far, it has been difficult to determine the conditions under which the genome data were generated. This study defined quality-related elements and profiled the FHIR genomic resource to report the experimental conditions and results produced in NGS genomic testing. Since our proposed method is based on ISO/TS 22692 and HL7 FHIR standards to interoperably share the quality-related data of clinical genomic testing, the app can communicate with any EMR/EHR systems that conform to these standards.

**Figure 6:**
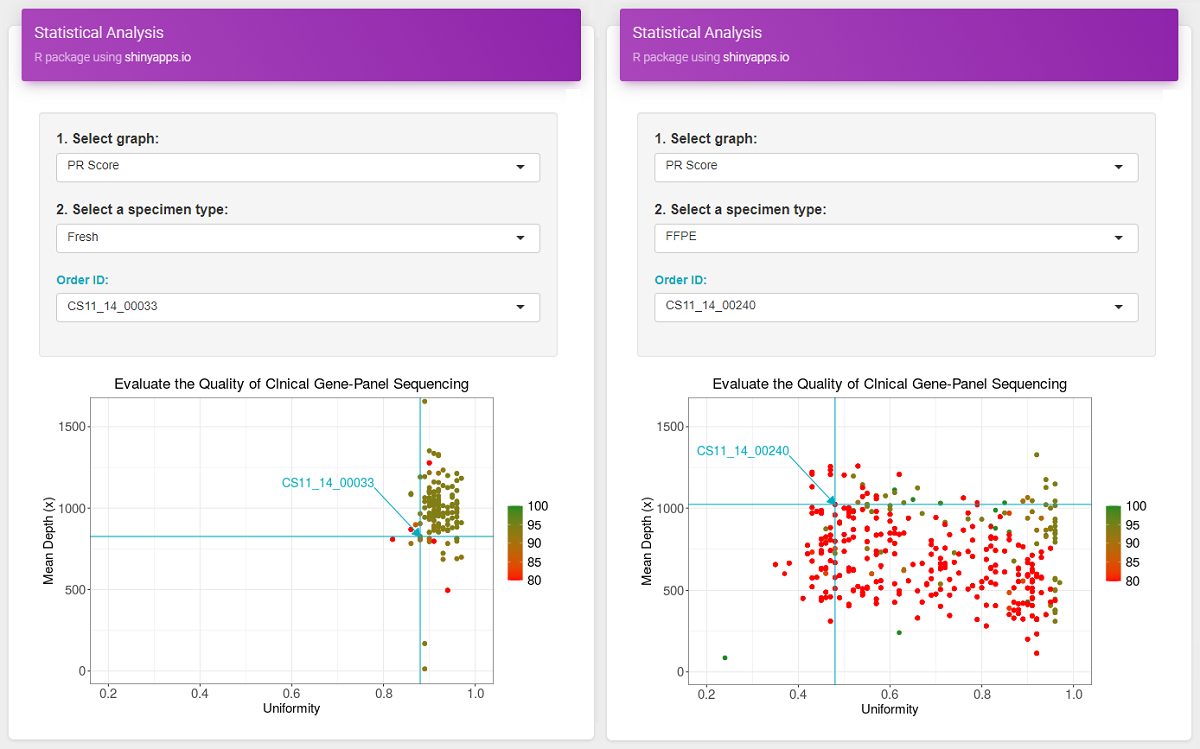
Example of comparative analysis based on specimen type using the NGS-QR app. FFPE: formalin-fixed paraffin-embedded; PR: pass rate.

Second, this study facilitated the verification of the quality status of each sample as experimental data accumulate. The quality of genome data is determined through comparative analysis based on the characteristics of NGS technology. As shown in Figure 6, the results from different types of specimens cannot be directly compared because this may lead to incorrect conclusions. Therefore, it is important to select the target groups and determine the performance of individual samples within them. The NGS-QR app allows users to select various experimental conditions and compare the produced data with the correct target group to check the exact data quality.

### Conclusions

This study successfully demonstrated how the quality information of clinical NGS genomic testing can be exchanged using a standardized method. As the demand for NGS genomic testing increases and genomic data accumulate, quality information can be used as reference material for improving the quality of testing. This approach can also motivate laboratories to perform diagnostic tests to provide high-quality genomic data.

## Abbreviations

API: application programming interface
EHR: electronic health record
EMR: electronic medical record
FDA: Food and Drug Administration
FFPE: formalin-fixed paraffin-embedded
FHIR: Fast Healthcare Interoperability Resource
ISO: International Organization for Standardization
MFDS: Ministry of Food and Drug Safety
NGS: next-generation sequencing
NGS-QR: Next-Generation Sequencing Quality Reporting
SMC: Samsung Medical Center
TLS: Transport Layer Security
UI: user interface
